# Biochemical outcome in metastatic prostate cancer patients following prostate-directed radiotherapy

**DOI:** 10.3332/ecancer.2024.1686

**Published:** 2024-03-26

**Authors:** Heba Maged Ayoub, Fifi Mostafa Elsayed, Maha Lotfy Zamzam, Ihab Mohamed Hassanin, Eman Essam Elsemary

**Affiliations:** Clinical Oncology Department, Faculty of Medicine, Suez Canal University, Ismailia 41522, Egypt

**Keywords:** biochemical outcome, low volume, metastatic prostate cancer, prostate-directed radiotherapy

## Abstract

**Background:**

The role of cytoreductive local radiotherapy (RT) in metastatic prostate cancer (mPCa) has recently been established. This study aimed to evaluate the biochemical outcome of local RT in mPCa.

**Methods:**

This randomised controlled phase III study was conducted at the Clinical Oncology Department, Suez Canal University Hospital. Eligible participants were de-novo or metachronous mPCa patients with Eastern Cooperative Oncology Group performance status of 0–2. Participants were randomised to receive either cytoreductive prostate-directed RT in addition to standard care or standard care alone. The conventional radiation schedule of 70 Gy/35 fractions or the hypofractionated schedule of 55 Gy/20 fractions were delivered. The primary endpoint was biochemical progression-free survival (BPFS), and the secondary endpoint was overall survival (OS). Survival and post-hoc analyses were performed using Cox regression and the Kaplan-Meier method with the log-rank test.

**Results:**

Between 23 November 2020 and 21 2022, 70 patients were enrolled in this study. Of them, 34 patients were assigned to the prostate RT group, and 29 patients were assigned to the control group. At a median follow-up of 12 months, the median BPFS has not been reached for the prostate RT group compared to 4.067 months for the control group (HR: 0.147, *p* < 0.001). Subgroup analysis showed that the median BPFS was statistically significantly correlated with low-volume (95% CI, 0.004 to 0.262, *p* = 0·001) and hormonal-sensitive metastatic disease (95% CI, 0.010 to 0.192, *p* < 0·001). The median OS was 16.33 months for the prostate-RT group compared to 11.33 months for the control group (HR: 0.313, *p* = 0.003).

**Conclusion:**

Prostate-directed RT improved BPFS and OS in mPCa patients, particularly in those with low volume and hormonal-sensitive disease.

**Trial Registration:**

This trial is registered on (27/4/2023), retrospectively registered with pactr.samrc.ac.za, PACTR202305854600529, URL: https://pactr.samrc.ac.za/TrialDisplay.aspx?TrialID=25510

## Background

Prostate cancer is the second most common male cancer and the fifth leading cause of male cancer mortality worldwide [1, 2]. Approximately 6% of newly diagnosed cases have metastatic disease [3, 4]. Despite tremendous advances in systemic treatment for hormone-naïve and castrate-resistant disease, metastatic prostate cancer (mPCa) remains incurable [5]. Androgen deprivation therapy (ADT) has long been the standard of care for the management of mPCa patients [6].

Local treatment of the primary prostate tumour in metastatic disease can disrupt the proliferation of malignant cells at metastatic sites, resulting in the regression of metastatic lesions [7]. Local cytoreductive treatment also disrupts the crosslinks between the primary tumour and metastatic foci, reducing the possibility of lineage adaptation and prolonging the time to castrate resistance [8]. Several studies have suggested that reducing tumour burden and the level of circulating tumour-derived factors through radical treatment can prevent the development of new metastases, prolong survival, and delay the progression of metastatic disease [9]. This has been explained through the tumour self-seeding hypothesis, which suggests that there are both primary tumour to metastases and metastases-to-metastases communication routes with the transfer of clones of cancer cells. The persistence of the primary tumour, even after systemic treatment, might provide a persistent uncontrollable source of malignant cells seeding new metastasis [10].

Cytoreductive radiotherapy (RT) directed to the primary tumour has currently been adopted as a new practice that can delay disease progression and improve survival outcomes in mPCa [11]. RT can be safely administered to both the primary tumour and the metastatic sites aiming for cytoreduction [12]. The stratification of mPCa patients based on metastatic burden into low-volume and high-volume metastatic disease has been associated with an increased probability of benefit from cytoreductive RT. Oligometastatic or low-volume metastatic disease is a favourable limited disease entity defined based on imaging studies between one and five osseous metastases confined to vertebral bodies and the pelvis without the existence of visceral metastases [13].

Combining cytoreductive prostate RT with standard hormonal and systemic treatment resulted in more sustained time to biochemical progression [11]. In addition, recent studies have reported a clinically meaningful overall survival (OS) benefit in oligo mPCa patients after receiving local RT [7, 14].

This study presents biochemical progression-free survival (BPFS) and OS in patients with mPCa treated with cytoreductive prostate-directed RT. We conducted further *post-hoc* analysis to correlate the volume of metastatic disease and fractionation scheme with the biochemical outcome following prostate-directed RT.

## Methods

### Study design and conduct

This phase III randomised controlled study was conducted at the Clinical Oncology Department of Suez Canal University Hospital in Ismailia governorate, Egypt, from November 2020 to 2022. The aim of the study was to evaluate the biochemical outcome of adding local cytoreductive prostate-directed RT to the standard of care in mPCa patients.

The study protocol was approved by the Research Ethics Committee at the institute, and all patients provided written informed consent before participating in the study. Patients had the right to withdraw from the study at any time. The authors take full responsibility for the accuracy, confidentiality of data, and adherence to the study protocol.

### Patients

The target sample size was calculated as 25 patients in each group. This provided 80% power at a 5% level of significance using the two-tailed unpaired *t*-test. Eligible patients were de-novo and metachronous mPCa patients with pathologically confirmed prostatic adenocarcinoma and a performance status of 0–2 according to the Eastern Cooperative Oncology Group performance scale. Patients with or without nodal involvement, low-volume mPCa (<4 bone metastases) or high-volume mPCa (≥4 bone metastases with at least 1 beyond the spine or pelvis, ± visceral metastases) according to the CHAARTED definition were included [15]. Patients who had a second malignancy, a performance status >2, underwent radical prostatectomy, received prior prostate RT/brachytherapy, or developed brain metastases were excluded. Seventy patients were enrolled at the institute from November 2020 through November 2022, of whom 63 were randomly assigned to receive either cytoreductive prostate-directed RT added to the standard of care (Prostate RT group) or the standard of care (control group).

### Randomization and masking

Patients were randomly allocated using computer-generated block randomisation with a block size of four, which equals two treatments with two patients per treatment. Stratification was not done at randomisation, and allocation was not concealed due to practical reasons related to the nature of the intervention.

### Intervention

All patients either received the standard of care androgen-deprivation therapy (luteinizing hormone–releasing hormone agonist ± bicalutamide) or underwent orchiectomy prior to randomisation. Prior systemic treatment with docetaxel, or novel androgen receptor antagonists were permitted. Prostate-directed RT was given as one of two regimens: either 70 Gy in 35 daily fractions of 2 Gy over 7 weeks, or 55 Gy in 20 daily fractions of 2.75 Gy over 4 weeks. Computerised tomography (CT) simulation was done in a supine position with an empty rectum and comfortably full bladder. Three-dimensional conformal planning was carried out, with the planning target volume including the prostate with a 10 mm margin, except posteriorly at the prostatic rectal interface (6 mm) and the proximal half of seminal vesicles. Pelvic nodal radiation was not included in this field. We delivered bone metastases-directed conformal 3D RT using the hypofractionated regimen 20 Gy/5 fractions in symptomatic patients only to provide palliation of painful bone metastases. Patients were followed up weekly during RT, then 1 month after finishing RT, and every 3 months for a year. Prostate-specific antigen (PSA) level was measured at baseline and routinely at every follow-up visit. The volume of metastatic disease was assessed through CT scans and skeletal scintigraphy and classified according to the CHAARTED definition. High-volume metastatic disease was defined as four or more bone lesions with one or more outside the vertebral bodies or pelvis, or visceral metastases, or both. Low volume metastatic disease was associated with less than four bone lesions not extending beyond the spine or pelvis.

### Endpoints

The primary endpoint was BPFS, defined as the time from randomisation to biochemical failure or progression. Biochemical failure was defined as nadir PSA plus 2 ng/ml according to Phoenix definition [16]. The secondary endpoint was OS, which was defined as the time from diagnosis to death. Patients who did not experience any event of interest were censored at the last follow-up.

### Statistical analysis

Statistical analysis was conducted using the Statistical Package for Social Science, version 28 (IBM Corporation, Armonk, NY, USA), on all collected data. Descriptive analysis was performed, with medians and interquartile ranges calculated for continuous variables, and means calculated for categorical variables. Fisher’s exact test and Pearson’s chi-square test were conducted to test for the significance of the association between categorical variables. We compared ADT duration and PSA median values using Mann–Whitney *U*-test. The survival analysis was performed and presented using Kaplan Meir and Cox Regression methods. The Log-Rank test was used to measure and compare survival differences between study groups. Hazard ratios with their 95% confidence intervals were calculated using multivariable Cox regression to determine the association between variables and survival outcomes. The univariate and multivariable *post-hoc* subgroup survival analysis was done through the Cox proportional hazards regression model using the Wilcoxon signed rank test for statistical significance testing. Furthermore, exploratory subgroup analysis was performed to compare survival outcomes according to different subgroups. Subgroup analyses were pre-specified for the baseline metastatic burden, the response to androgen deprivation, and the received RT regimen. All statistical tests were two-sided, and *p*-values of <0.05 were considered statistically significant.

## Results

**Figure 1.**
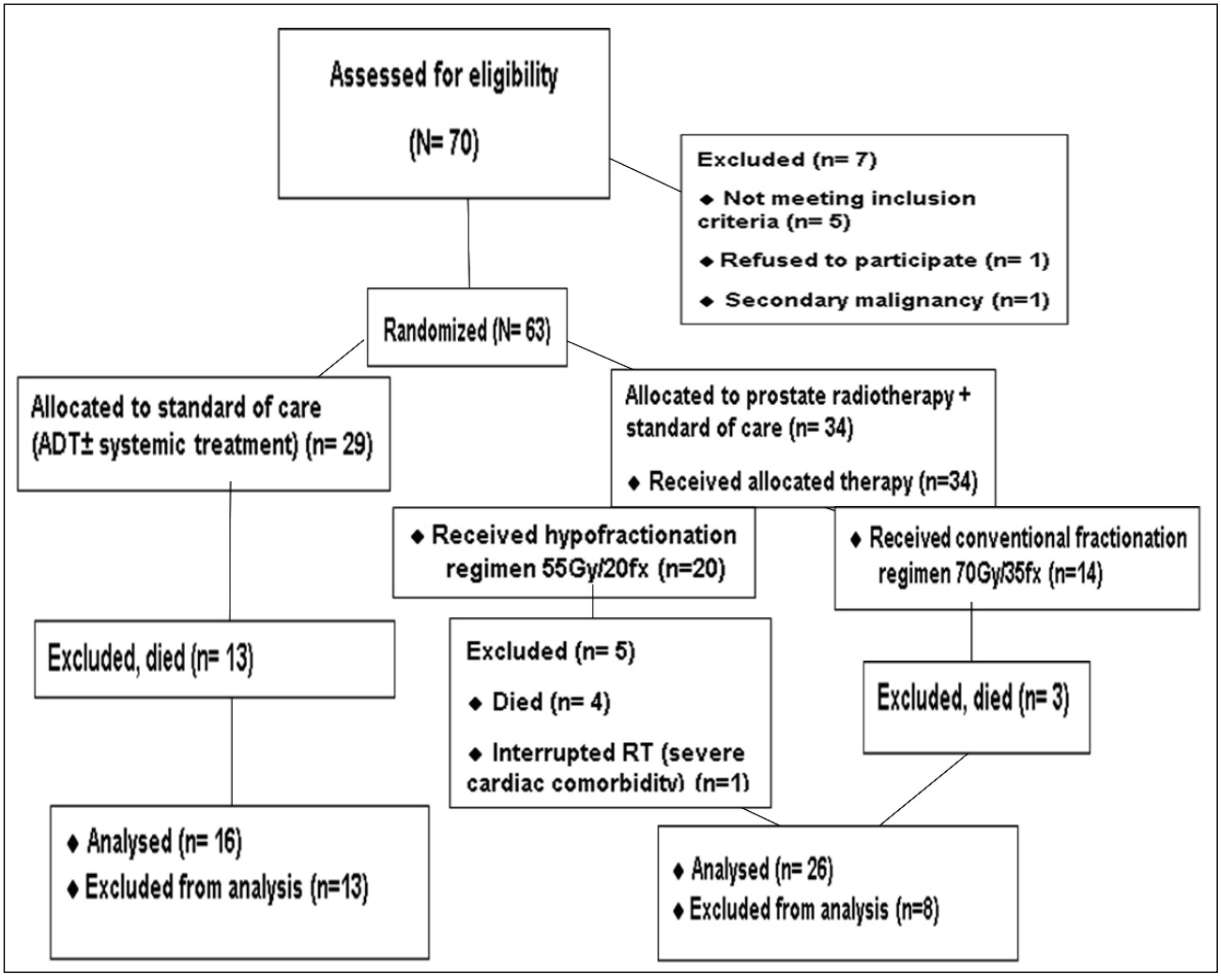
CONSORT diagram of the study.

We enrolled 70 patients with mPCa between 23 November 2020 and 21 2022. Of these, 63 patients were randomly assigned to either the standard of care (control group, *n* = 29) or the standard of care plus RT (RT group, *n* = 34) (Figure 1).

The distribution of sociodemographic and clinicopathological characteristics was mostly balanced between the study groups. However, there were statistically significant differences in nodal involvement and palliative bone-directed radiation treatment between both groups. The median age of the patients was 70 years (IQR 66–75). The most common histopathological subtype was acinar adenocarcinoma (88.9%). The most frequently observed histological grade was Gleason score 8–10 (74.6%). T3, N0 and M1b were the most frequent stages. Nodal involvement (N+) was statistically significantly higher among the RT group than the control group (*p* = 0.035). High-volume metastatic disease was present in 63.5% of patients in both groups. Most patients had de-novo (synchronous) metastatic disease (85.7%). The majority of patients had a performance score of ≤1 (66.7%). Palliative bone RT was received among patients in the control group statistically significantly higher than patients in the prostate RT group (*p* < 0.001) (Table 1).

The median PSA was 100 ng/ml (IQR 41–270), and the median duration of ADT was 3.5 months (IQR 2.25–9). Most patients received combined luteinising hormone-releasing hormone (LHRH) agonist and bicalutamide (71.4%). Most patients in both groups were castrate-sensitive (68.3%). Docetaxel was received by 25.4% of patients, whereas 22.2% received abiraterone acetate (Table 1).

**Figure 2.**
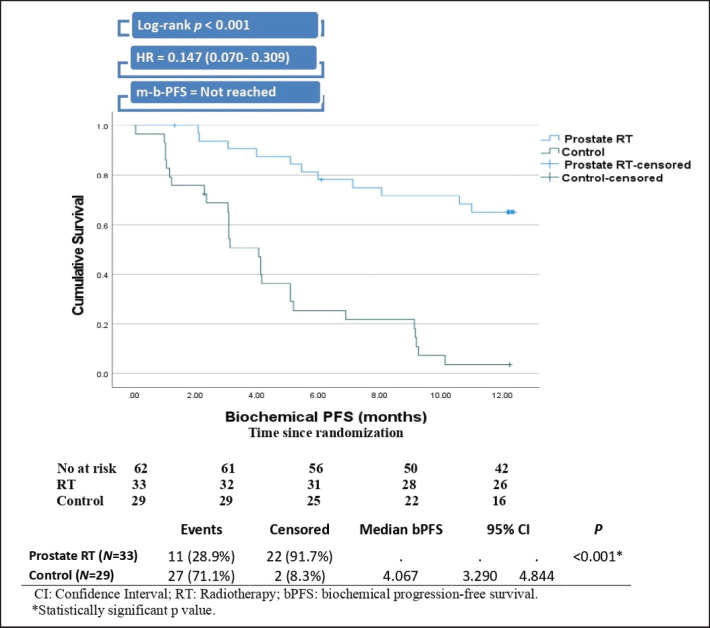
Kaplan–Meier curve and plot of BPFS.

After a median follow-up of 12 months (IQR 10–14), the RT group demonstrated significantly longer BPFS than the control group (not reached versus 4.07 months, Log-rank *p* < 0.001. The risk of biochemical progression among patients in the RT group was 85.3% lower than the risk among the control group, with a hazard ratio of 0.147 (Figure 2).

**Figure 3.**
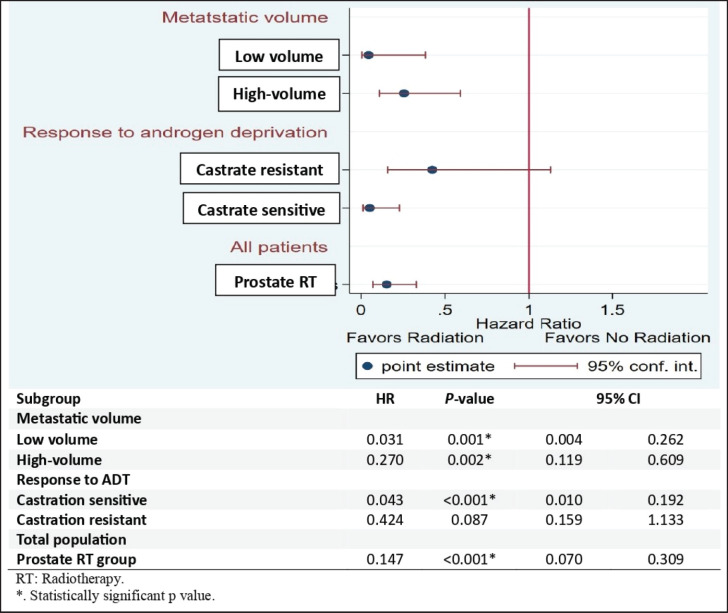
Forest plot for subgroup analysis of BPFS.

Subgroup analysis revealed that low-volume metastatic patients in the RT group had a significantly lower risk of biochemical progression than those in the control group (HR: 0.031, 95% CI 0.04–0.262;* p* = 0.001). Additionally, high-volume metastatic patients in the RT group had a significantly lower risk of biochemical progression than their respective counterparts in the control group (HR: 0.270, 95% CI 0.19–0.609;* p* = 0.002). Metastatic castration-sensitive patients in the RT group had a statistically significantly lower risk of biochemical progression than metastatic castration-sensitive patients in the control group (HR: 0.043, 95% CI 0.010–0.192; *p* < 0.001). Overall, patients in the RT group had lower risk of biochemical progression than patients in the control group (HR: 0.147, 95% CI 0.070–0.309;* p* < 0.001) (Figure 3).

Multivariable *post-hoc* analysis for BPFS showed that the median BPFS was significantly associated with the low volume subgroup (adjusted HR: 0.140, 95% CI 0.023–0.861;* p* = 0.034) and castration-sensitive subgroup (adjusted HR: 0.044, 95% CI 0.008–0.251; *p* < 0.001), but not significantly associated with the hypofractionated regimen (adjusted HR: 0.434, 95% CI 0.108–1.753;* p* = 0.241) (Table 3).

Among subgroups receiving prostate RT, the median BPFS was significantly better for the low-volume metastatic subgroup than for the high-volume subgroup (not reached versus 10.6 months, log-rank *p* = 0.026). The metastatic castration-sensitive/naïve subgroup had significantly better BPFS than the castration-resistant subgroup (not reached versus 6 months, log-rank *p* < 0.001). The median BPFS was not significantly different between both fractionation regimens (*p* = 0.568) (log-rank *p* = 0.568) (Table 2).

**Figure 4.**
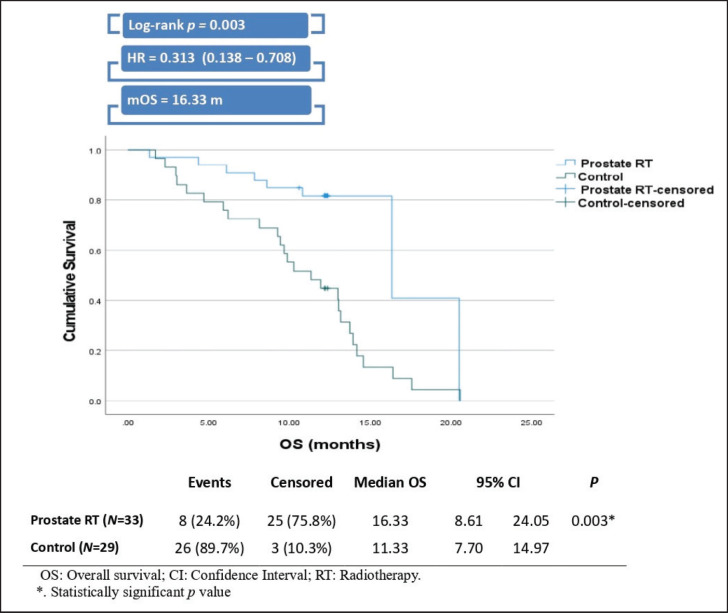
Kaplan-Meier curve and plot of OS.

The prostate RT group demonstrated a significantly greater median OS than the control group, with a median of 16.33 months compared to 11.33 months for the control group (Log-rank *p =* 0.003). Patients in the prostate RT group also had a 68.7% lower risk of death compared to those in the control group, with a hazard ratio of 0.313 (Figure 4).

**Figure 5.**
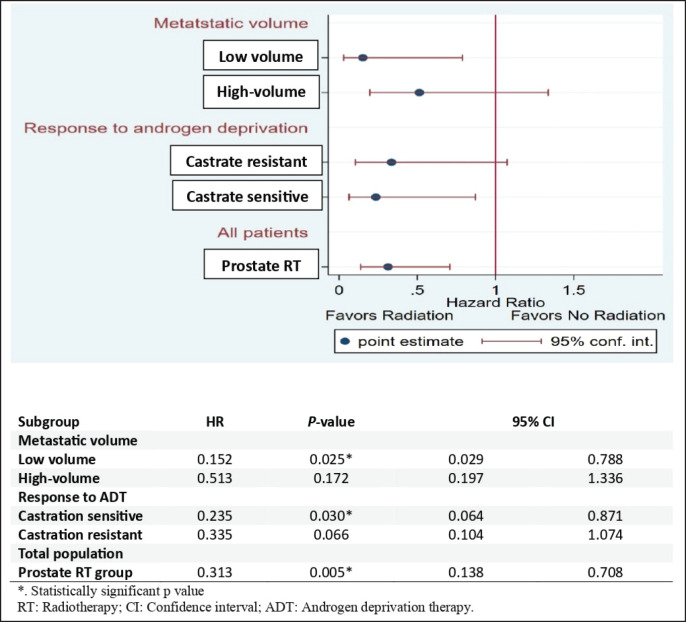
Forest plot for subgroup analysis of OS.

Subgroup analysis for OS indicated that low-volume metastatic patients in the RT group had a significantly lower risk of death than those in the control group (HR: 0.152, 95% CI 0.029–0.788;* p* = 0.025). Additionally, metastatic castrate-sensitive patients in the RT group had a significantly lower risk of death than those in the control group (HR: 0.235, 95% CI 0.064–0.871;* p* = 0.030). Overall, the prostate RT group had a significantly lower risk of death than the control group, with a hazard ratio of 0.313 (HR: 0.313, 95% CI 0.138–0.708;* p* = 0.005) (Figure 5).

Our regression analysis for OS did not show any statistically significant differences between low-volume and high-volume metastatic subgroups, castration-sensitive and castration-resistant subgroups, and conventional and hypofractionation regimens.

The multivariable *post-hoc* analysis for OS indicated that OS was not significantly associated with the low metastatic volume subgroup (adjusted HR: 0.206, *p* = 0.150), castration sensitive subgroup (adjusted HR: 0.585, *p* = 0.533) and hypofractionation subgroup (adjusted HR: 0.855, *p* = 0.853) (Table 4).

## Discussion

We conducted this randomised controlled study aiming to evaluate the survival benefits of adding cytoreductive prostate RT to the standard of care in mPCa patients. First, regarding patients’ characteristics, our study population had a median age of 70 years old. Most patients had concurrent comorbidities (65.1%).

Most patients were also presented with T3+ disease (67.1%), N0 (61.9%) and M1b (77.8%). Nodal involvement was incidentally higher among the prostate RT group (50% versus 24.1%). Bone metastases were detected in 69.8% whereas visceral metastases were detected in 14.3% of patients. Most patients had a high Gleason score of 8–10 (74.6%) and the performance score of 1 (65.1%). Additionally, high metastatic volume disease was stratified in 63.5% whereas low metastatic volume was classified in 36.5% of patients. Docetaxel was received by 25% of patients and second line anti-androgens were received by 27% of patients. Palliative bone-directed RT was received by 54% of patients. Of note, palliative bone directed RT was delivered to a higher number of patients in the control group than the prostate RT group due to their more symptomatic painful bone metastases (86.2% versus 26.5%).

In comparison, arm H of the STAMPEDE randomised controlled phase III trial conducted by Parker *et al* [17] treated 1,032 metastatic hormone-naïve prostate cancer patients with prostate RT at 117 hospitals across Switzerland and the UK. Despite the large sample size difference (2,061 versus 63 patients), most of their patients’ characteristics were similar to ours, with a median population age of 68 years and 56.5% of their patients having comorbidities. However, their patients were presented with more T3+ disease (90%) and M1b (89%) but with more N1 disease (64%) and fewer N0 disease (36%). Bone metastases were found in 89% of their patients, while visceral metastases were detected in 9.5%. Most patients had a Gleason score of 8–10 (82.5%), and a performance score of 0 in 71%. Low metastatic burden was stratified in 42.5% whereas high metastatic burden in 57.5% of their patients. Docetaxel was received by 18% of their population. However, there was no data regarding any second line anti-androgen received. Palliative metastases-directed RT was not yet received by their patients [17].

The HORRAD study by Boevé *et al* [14] randomised 432 mPCa patients with slightly different inclusion criteria (PSA >20 ng/ml and bone metastases) and included a larger sample size to determine if there was a survival benefit from external beam radiation therapy (EBRT) to the primary prostate in patients diagnosed with mPCa. Their patients’ characteristics were comparable to ours, with a median age of 67 years and most of their patients similarly having T3+ disease (84%). However, they had a better performance score than our patients, PS 0 (84.5%), and a higher proportion of their patients received docetaxel (44%). Second-line anti-androgens were received by only 8% of their patients, and palliative metastases-directed RT was not yet received by their patients [14].

Regarding patient stratification, our study stratified mPCa patients according to their metastatic disease volume into high-volume and low-volume metastatic disease. Boevé *et al* [14] differently subdivided their patients into <5, 5–15, or more bone lesions based on skeletal scintigraphy. Similar to our study, Boevé *et al* [14] underwent no stratification at the time of randomisation. In contrast, Parker *et al* [17] stratified patients at randomisation based on their metastatic burden into low and high burden disease. Additionally, they stratified patients based on their age, nodal involvement, performance score, planned hormonal treatment, analgesic use, and docetaxel administration [17].

Regarding the radiation dose, we prescribed the same conventional regimen 70 Gy in 35 fractions over 7 weeks which was prescribed by Boevé *et al* [14]. In this study, we used the same hypofractionated regimen (55 Gy in 20 daily fractions over 4 weeks) prescribed by Parker *et al* [17]. This regimen offered several advantages, including shorter overall treatment duration, cost-effectiveness, and patient convenience. The radiation dose used in our study was lower than the current doses used in clinical practice for localised prostate cancer. Higher radiation doses have been studied in dose-escalating studies, but the benefits for patient survival were doubtful. Moreover, escalated doses come with a risk of increased gastrointestinal and genitourinary toxicities in metastatic patients [18]. Therefore, the two more convenient schedules (70 Gy/35fx and 55 Gy/20fx) were permitted in this study.

The regression analysis for biochemical and survival outcomes has demonstrated the significant impact of cytoreductive prostate-directed RT on BPFS in mPCa patients. Parallel to our findings, Boevé *et al* [11] reported that the median BPFS was better in the prostate RT group compared to the control group (*p* = 0.02). Our prespecified subgroup analysis revealed that prostate RT improved BPFS in low metastatic volume (HR: 0.031, *p* = 0.001), high metastatic volume (HR: 0.270, *p* = 0.002), and metastatic castration-sensitive disease (HR: 0.043, *p* < 0.001). Meanwhile, Burdett *et al* [19] showed that there was an improvement in BPFS with prostate RT (HR: 0.74, *p < 0.001*).

In addition, our study found an OS benefit resulting from adding prostate RT to standard ADT in mPCa patients (median OS = 16.33 versus 11.33, *p* = 0.003). However, the short OS of patients in the standard of care group could be also attributed to the higher age group in this population where 86.2% of patients are older than 65 years old. In addition, patients in the control group had higher incidence of comorbidities and worse performance status than patients in the RT group. Conversely, Boevé *et al* [11] found no significant OS difference between the prostate RT group and the control group. However, they reported better OS, particularly in patients with five or fewer bone metastases treated with prostate RT (HR: 0.68) [11].

Contrary to our findings, Parker *et al* [17, 18] prostate RT did not significantly impact OS in the total cohort at a median follow-up of 61.3 months (HR: 1.00, *p* = 0.974). Parker *et al* [17, 18] consistently found, through subgroup analysis, that prostate RT continued to offer a significant OS benefit in the low metastatic burden subgroup after a median follow-up of 61.3 months (HR: 0.64, *p* < 0.001). Their exploratory subgroup analysis, corresponding to our data, indicated that prostate RT did not significantly improve OS for patients in the high metastatic burden subgroup (HR: 1.11, *p* = 0.164) [17, 18].

Sooriakumaran *et al* [20] discovered that patients who received RT in addition to ADT had a three times lower mortality rate than patients who received ADT alone. Culp *et al* [21] published that mPCa patients who received prostate brachytherapy had a better 5-year OS compared to patients who underwent no local therapy (5-year OS: 52.6% versus 22.5%; *p* < 0.001). Antwi and Everson [22] suggested that local therapy for primary disease in mPCa, including brachytherapy, improved survival outcomes with a 57% lower mortality risk. They also found that the mortality risk was higher for visceral disease (M1c) and bone-restricted disease (M1b) as compared to node-positive disease (M1a) [22].

Satkunasivam *et al* [23] concluded that mPCa patients who received prostate intensity-modulated radiation therapy (IMRT) had a 62% reduction in mortality risk compared to the control group. Rusthoven *et al* [24] concluded that mPCa patients who received prostate RT in addition to ADT had a statistically significant absolute 5-year OS benefit of 16% compared to ADT alone (HR: 0.62, *p* < 0.0001) [24]. Contrary to our findings, Satkunasivam *et al* [23] and Parikh *et al* [25] found that conformal radiation therapy was not associated with improved OS.

Leyh-Bannurah *et al* [26] concluded that prostate-directed RT, when combined with ADT, resulted in better OS in mPCa compared to ADT alone. Löppenberg *et al* [27] found that the 3-year OS was higher in patients who received local therapy, including prostate RT, compared to those who received no local therapy (69% versus 54%). Similar to our findings, Löppenberg *et al* [27] concluded that patients with a low tumour volume tend to benefit greatly from RT. Parikh *et al* [25] also found that patients who received local therapy, including prostate IMRT, had improved 5-year OS (45.7% versus 17.1%) compared to those who did not receive local therapy. They concluded that prostate RT remained significantly associated with OS (HR: 0.35, *p*  <  0.01) [25].

Morgan *et al* [2,8] found that prostate RT was associated with improved OS (HR: 0.59, *p*  =  0.0001). The 2 and 5-year OS rates were 74.5% and 41.1%, respectively, for those receiving prostate RT and 53.1% and 25.0%, respectively, for those not receiving RT. Morgan *et al* [28] reported a median OS of 47.4 months for patients who received prostate RT compared to 26.3 months for those who did not. Thereafter, Burdett *et al* [19] conducted the STOPCAP M1 meta-analysis and reported opposite findings to ours, showing no improvement in OS with prostate RT (HR: 0.92, *p* = 0.195). However, they did report a 7% improvement in 3-year OS for patients with low-volume disease [19].

Concerning palliative bone-targeted RT, we delivered palliative radiation to symptomatic patients as suggested by Phillips *et al* [29] who focused on the survival advantage of further treatment of all oligometastases. However, our results didn’t demonstrate OS or BPFS survival benefits resulting from the addition of palliative bone-directed RT. This could be more obvious for the control patients who were treated more with bone-targeted RT without reflecting upon OS or BPFS outcomes.

There were some limitations in our study. Firstly, the small sample size may affect the generalisability of our results. Secondly, the short duration of follow-up may have underestimated the potential benefits of prostate RT and could have failed to detect late effects that may take years to develop. The lack of experience with IMRT in our centre was another limitation. Additionally, the precise correlation between the volume of disease and the degree of benefit from local RT remains unclear. Finally, we did not require the use of prostate specific membrane antigen positron emission tomography (PSMA-PET) due to resource limitations, even though PSMA-PET has shown higher sensitivity for the detection of metastatic deposits than conventional imaging [30, 31].

## Conclusion

Prostate-directed RT in mPCa patients is a new practice that can improve response, survival and retard biochemical progression. In particular, cytoreductive RT of the prostate should be considered as an adjunct to the standard AD and systemic treatment, especially in low-volume and hormone-sensitive metastatic disease. Despite the few numbers of patients enrolled in this study, our results were quite similar to previous randomised controlled studies published demonstrating that in patients with low-volume disease, prostate RT has improved biochemical and OS outcomes. The main differences are that our analysis showed better outcomes additionally in a high volume disease, differently compared to STAMPEDE and HORRAD studies. Importantly, defining low volume and oligometastatic disease, determining the preferred imaging modality, selecting the optimal RT technique, dose, and timing in the context of upfront combination chemo-hormonal treatment remains questionable.

Further randomised controlled trials with larger sample sizes and multicentre designs are necessary to refine the definition of oligometastatic disease and identify the optimal radiation dose, timing, and technique of prostate-directed RT in the metastatic setting. Future prospective studies will provide valuable insights for refining selection criteria of mPCa patients for local RT.

## List of abbreviations

BPFS, Biochemical progression-free survival; IMRT, Intensity-modulated radiation therapy; mPCa, Metastatic prostate cancer; OS, Overall survival; PSA, Prostate specific antigen; RT, Radiotherapy.

## Conflicts of interests

The authors declare no competing interests. The authors have no affiliations or involvement with any organisation or entity with a financial interest. This includes educational grants, membership, employment, honoraria, consultancies, stock ownership, expert testimony, patent licensing arrangements, or non-financial interest in the subject matter or materials discussed in this manuscript. All authors agree with the contents of the manuscript and there is no financial interest to report. We certify that the submission is original work and is not under review at any other publication.

## Funding

This research received no specific grant from any funding agency in the public, commercial, or not-for-profit sectors.

## Consent for publication

Not applicable.

## Ethics approval and consent to participate

Ethical approval for this study was obtained from the Research Ethics Committee at the Faculty of Medicine, Suez Canal University (approval number: 4492) on 1 March 2020. All participants provided informed consent prior to participation in the research, and the study was conducted in accordance with the principles of the declaration of Helsinki.

## Availability of data and materials

Data supporting this research findings were collected from registries and medical records at the Department of Clinical Oncology, Suez Canal University Hospital. Datasets supporting the conclusion of this article are attached with the article.

## Author contributions

All authors contributed to the study conception and design. Material preparation, data collection and analysis were performed by HA, EE, FE, IH and MZ. The first draft of the manuscript was written by HA and all authors revised and commented on previous versions of the manuscript. All authors read, critically reviewed, and approved the final manuscript.

## Figures and Tables

**Table 1.** Sociodemographic, clinical and treatment characteristics among study groups.

Variables	Prostate RT group *n* = 34 (54%)	Control group *n* = 29 (46%)	*p*-value
Age (years) <60	2 (5.9%)	1 (3.4%)	0.432^f^
60–65	8 (23.5%)	3 (10.3%)	
>65	24 (70.5%)	25 (86.2%)	
Median (IQR)	68 (64–72)	73 (68.5–79.5)	
Histological subtype Acinar adenocarcinoma	32 (94.1%)	24 (82.8%)	0.233^f^
Others	2 (5.9%)	5 (17.2%)	
Gleason score ≤7	12 (35.3%)	4 (13.8%)	0.081^f^
8–10	22 (64.7%)	25 (86.2%)	
Grade group 1–2	8 (23.5%)	2 (6.9%)	0.128^f^
3	4 (11.8%)	2 (6.9%)	
4–5	22 (64.7%)	25 (86.2%)	
***T*** (Stage) T2	13 (38.2%)	8 (27.6%)	0.371^*χ*^2^^
≥ T3	21 (61.8%)	21 (72.4%)	
***N*** (Stage) N0	17 (50%)	22 (75.9%)	0.035* ^*χ*^2^^
N+	17 (50%)	7 (24.1%)	
***M*** (Stage) M1a	3 (8.8%)	0 (0%)	0.349^f^
M1b	25 (73.5%)	24 (82.8%)	
M1c	6 (17.6%)	5 (17.2%)	
Metastatic volume High volume	18 (52.9%)	22 (75.9%)	0.060^*χ*^2^^
Low volume	16 (47.1%)	7 (24.1%)	
Timing of metastases De-novo	27 (79.4%)	27 (93.1%)	0.160^f^
Metachronous	7 (20.6%)	2 (6.9%)	
Performance status 0–1	25 (73.5%)	17 (58.6%)	0.211^*χ*^2^^
2	9 (26.5%)	12 (41.4%)	
Prior ADT duration, median (IQR)	4.25 (2.5–9)	2.8 (1.9–8)	0.073
Baseline PSA, median (IQR)	89.5 (32–163.8)	100 (58.3–375)	0.098
PSA at RT timing, median (IQR)	9.5 (0.7–100)	10 (0.7–57.5)	0.818^*χ*^2^^
Hormonal treatment LHRH agonist only	3 (8.8%)	5 (17.2%)	0.453^f^
Bicalutamide only	6 (17.6%)	3 (10.3%)	0.488^f^
Combined	25 (73.5%)	21 (72.4%)	0.921^*χ*^2^^
Type of castration Medical	28 (82.4%)	23 (79.3%)	0.759^*χ*^2^^
Surgical	6 (17.6%)	6 (20.7%)	
Response to ADT Castration naïve/sensitive	24 (70.6%)	19 (65.5%)	0.666^*χ*^2^^
Castration resistant	10 (29.4%)	10 (34.5%)	
Number of treatment lines			
1	22 (64.7%)	17 (58.6%)	
2	7 (20.6%)	10 (34.5%)	0.418^f^
>2	5 (14.7%)	2 (6.9%)	
Prior treatment			
Docetaxel	7 (20.6%)	9 (31%)	0.342^*χ*^2^^
Abiraterone acetate	10 (29.4%)	4 (13.8%)	0.224^f^
Enzalutamide	2 (5.9%)	1 (3.4%)	1.000^f^
Palliative bone RT	9 (26.5%)	25 (86.2%)	<0.001* ^*χ*^2^^
Bisphosphonates Zoledronic acid	25 (73.5%)	27 (93.1%)	0.124^f^
Denosumab	4 (11.8%)	0 (0%)	
Both	1 (2.9%)	0 (0%)	
None	4 (11.8%)	2 (6.9%)	
Comorbidities	12 (35.3%)	19 (56.5%)	0.164 ^*χ*^2^^

f Fisher’s exact test,

*χ* ^2^ Chi-square test,

* Statistically significant *p* value

ADT: Androgen deprivation therapy, IQR: Interquartile range, RT: radiotherapy

**Table 2.** BPFS among subgroups receiving prostate RT.

Subgroup	BPFS events *N* (%)	Censored N (%)	Median BPFS (months)	95% CI		Log-rank *p*-value
				Lower	Upper	
Metastatic volume						
Low volume (*n* = 15)	2 (18.2%)	13 (59.1%)	.			0.026*
High-volume (*n* = 18)	9 (81.8%)	9 (40.9%)	10.600	.	.	
Response to ADT						
Castration sensitive (*n* = 23)	2 (18.2%)	21 (95.5%)	.	.	.	<0.001*
Castration resistant (*n* = 10)	9 (81.8%)	1(4.5%)	6.000	3.417	8.583	
Fractionation regimen						
Hypofractionation (*n* = 20)	6 (54.5%)	14 (63.6%)	.	.	.	0.568
Conventional (*n* = 13)	5 (45.5%)	8 (36.4%)	.	.	.	

* Statistically significant *p* value

ADT: Androgen deprivation therapy, bPFS: biochemical progression-free survival, CI: Confidence interval

**Table 3.** Univariate and multivariable subgroup analysis for BPFS in prostate RT group.

Subgroup	Univariate				Multivariable			
	HR	*p*	95% CI		HR	*p*	95% CI	
			Lower	Upper			Lower	Upper
Metastatic volume								
Low volume	0.205	0.043*	0.044	0.955	0.140	0.034*	0.023	0.861
High-volume	1				1			
Response to ADT								
Castration sensitive	0.060	<0.001*	0.013	0.286	0.044	<0.001*	0.008	0.251
Castration resistant	1				1			
Fractionation regimen								
Hypofractionation	0.708	0.570	0.215	2.331	0.434	0.241	0.108	1.753
Conventional	1				1			

* Statistically significant *p* value

HR: Hazard ratio, ADT: Androgen deprivation therapy

**Table 4.** Univariate and multivariable subgroup analysis for OS in prostate RT group.

Subgroup	Univariate				Multivariable			
	HR	*p*	95% CI		HR	*p*	95% CI	
			Lower	Upper			Lower	Upper
Metastatic volume								
Low metastatic volume	0.185	0.118	0.022	1.538	0.206	0.15	0.024	1.77
High metastatic volume	1				1			
Response to ADT								
Castration sensitive	0.442	0.318	0.089	2.193	0.589	0.533	0.111	3.115
Castration resistant	1				1			
Fractionation regimen								
Hypofractionation	0.609	0.544	0.123	3.021	0.855	0.853	0.163	4.505
Conventional	1				1			

HR: Hazard ratio, CI: Confidence interval
